# Nanomaterial Production from Metallic Vapor Bubble Collapse in Liquid Nitrogen

**DOI:** 10.3390/nano13132021

**Published:** 2023-07-07

**Authors:** Chen Li, Ruoyu Han, Jingran Li, Yuchen Cao, Wei Yuan, Qifan Li

**Affiliations:** 1 State Key Laboratory of Explosion Science and Technology, Beijing Institute of Technology, Beijing 100081, China; 3120205735@bit.edu.cn; 2 State Key Laboratory of Mechatronics Engineering and Control, Beijing Institute of Technology, Beijing 100081, China; 1120201015@bit.edu.cn (J.L.); 3120211508@bit.edu.cn (Y.C.); 3120211546@bit.edu.cn (W.Y.); 3 School of Materials and Energy, University of Electronic Science and Technology of China, Chengdu 610054, China

**Keywords:** electrical explosion of wires, liquid nitrogen, bubble dynamics, particle formation mechanism

## Abstract

Nanomaterials with unique structural and properties can be synthesized by rapid transition of the thermodynamic state. One promising method is through electrical explosion, which possesses ultrafast heating/quenching rates (d*T*/d*t*~10^9^ K/s) of the exploding conductor. In this study, experiments were performed with fine metallic wire exploding in liquid nitrogen (liq N2, 77 K) under different applied voltages. For the first time in the literature, the physical image of the electrical explosion dynamics in liq N2 is depicted using electro-physical diagnostics and spatial-temporal-resolved photography. Specifically, the pulsation and collapse processes of the vapor bubble (explosion products) have been carefully observed and analyzed. As a comparison, an underwater electrical explosion was also performed. The experimental results suggest that the vapor bubble behavior in liq N2 differs from that in water, especially in the collapse phase, characterized by secondary small-scale bubbles in liq N2, but multiple bubble pulses in water; correspondingly, the products’ characteristics are discrepant.

## 1. Introduction

Electrical explosion of wires (EEW) is a unique approach for the one-step synthesis of particles or coatings with nanoscale structural characteristics, characterized by ultrafast heating and quenching rates (d*T*/d*t*~10^10^ K/s) of the sample [1,2]. During an EEW process, a fine wire undergoes rapid phase transitions due to Joule heating by a large pulsed current, dispersing into the gaseous state or a mixture of vapor and small droplets. This is usually accompanied by ionization and plasma radiation with a temperature of 10^4^ K or higher [3,4]. Later, the electrical explosion products expand into the surrounding medium at a high velocity (initial velocity of several km/s), arousing shock waves and turbulent flows [5,6]. Fluid instabilities soon develop, promoting interactions such as energy transfer and chemical reactions between the hot explosion products and the cold ambient medium, which quenches the products into nanomaterials. This simple method can be used to prepare nanomaterials of pure metals, oxides, and nitrides by exploding the wire in various working ambient media [7,8,9].

The parameters of the ambient medium significantly influence the microscopic characteristics of EEW-prepared nanomaterials. Atomized products collide with cold atoms/molecules of the ambient medium and undergo nucleation, condensation, and coagulation processes. Thermo-physical properties such as pressure and thermal conductivity generally dominate these processes in gaseous media [10,11]. The particle dimension is inversely proportional to the saturation ratio *S*, defined as the ratio between the pressure exerted by the vapor and the saturation vapor pressure [12]. A faster cooling rate leads to a higher level of *S* due to the reduction of saturation vapor pressure, resulting in nanoparticles of a lower dimension. Sindhu et al. found that the geometric mean size was smallest in helium ambiance compared to argon and nitrogen gas in both experiment and simulation due to the higher thermal conductivity (cooling rate) of helium [12]. However, the rapid phase transition of the exploding wire induces surface flashover, which causes incomplete vaporization/atomization, increasing the inhomogeneity of the explosion products [13,14]. Additionally, the development of electro-thermal instability, characterized by periodic striations, can increase the size range of the nanoparticles [15].

Research on exploding wires in liquid media, such as water and liquid nitrogen, has been ongoing for about 15 years [16]. It is believed that in electrically insulating liquids, a higher overheating factor (deposited energy to atomization enthalpy) and uniform heating of the wire lead to the formation of finer particles [17]. Additionally, liquids possess greater thermal conductivities than gaseous media, cooling down the explosive products rapidly [18]. Comparing the synthesis of nanopowders by EEW in air and water, Cho et al. found that the water medium produced smaller particles due to the higher deposited energy expansion volume and quick cooling of the particles [16]. While reaction synthesis was developed in a liquid nitrogen environment, due to the high activity of atomized metals, the ambient media not only determines the chemical composition of the compound but also has a prominent influence on nanoparticle formation, such as the high thermal conductivity and extremely low temperature (77 K) of liq N2. The nucleation and growth mechanism of synthesis nanoparticles in liquid via the EEW method has different perspectives. Shutesh et al. proposed that particle formation came from the nucleation of the cooling of the overheated metal vapor by collision with water molecules, followed by growth through coagulation via Brownian movement, which followed the common formation mechanism in air explosions [19]. Peng et al. suggested that the bubble pulse in the liquid explosion should be considered as it contributes to the size distribution feature and illustrated that the dynamics of nucleation and condensation processes were discrepant between the surface and the interior of the bubble, which were relevant to the Leidenfrost phenomenon [20]. Furthermore, there still exists controversy in the particle formation mechanism in liquid explosions. More elaborate visual evolution processes are needed when considering the dynamics of the bubble pulse, as existing investigations are insufficient. Finally, little attention has been paid to the influence of inherent thermal characteristics of liquid media, such as water and liq N2, on the final products.

In this study, we conducted experiments to explore the formation of nanoparticles in liquid media using a single copper wire exploding in liquid nitrogen at three different applied voltages, each corresponding to a different initial stored energy. As a comparison, we also conducted experiments using water as the medium. To analyze and visualize the physical and dynamic processes, we utilized electrical diagnosis and high-speed photography technology, with a particular focus on the bubble pulse that may be involved in the nucleation process. Additionally, we characterized the samples’ morphology and crystallographic characteristics using SEM, HRTEM, and XRD devices. Finally, we conducted a coalition analysis of the electrical explosion process and explosive products to gain a more in-depth understanding of the underlying physical and chemical phenomena.

## 2. Experimental Setup

### 2.1. Electrical Explosion Device and Diagnosis Methods

**Figure 1:**
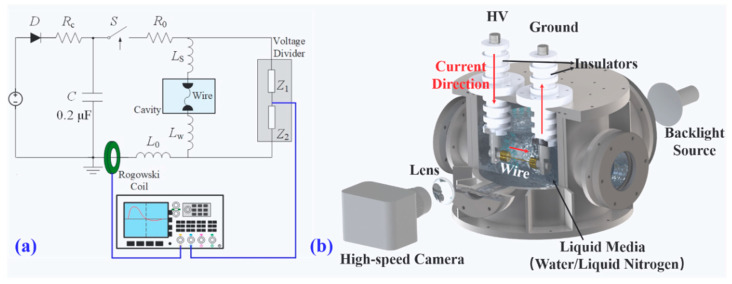
Schematics of the experimental setup and configurations: (**a**) circuit diagram and (**b**) cross-section view of the chamber.

Figure 1a illustrates the circuit diagram used in the study, where a 0.2 µF pulse capacitor was charged up to a certain positive voltage by a DC power supply. A coaxial-triggered switch connected to the capacitor was used to control the pulse current passing through the load wire, producing a microsecond timescale 10–20 kA current pulse to drive the electrical explosion. Additional details on the equipment can be found elsewhere [2,6,15]. Figure 1b provides a schematic diagram of the exploding cavity, which was made of stainless steel and contained a couple of insulators and copper electrodes. The cavity was filled with liquid media (tap water or liquid nitrogen), and the exploding wire was completely immersed in it.

For electrical parameter diagnosis, the discharge voltage *u* and current *i* were measured with a PVM-5 probe (bandwidth of 80 MHz) and Pearson 101 coil (bandwidth of 4 MHz), respectively. The resistive load voltage *u*_R_ and the energy deposition *E*_d_ were estimated using Equations (1) and (2) as

(1) $$u_{R}\left(t\right)\;\approx u-L_{s}\frac{di}{dt},$$

(2) $$\mathrm{and}\;E_{d}=\int \;u_{R}\left(t\right)i\left(t\right)dt,$$

where *L*_s_ refers to the inductance of the wire and its holder. The correctness of voltage and current measurement was examined by conducting the pulsed current with a low inductance (nH level) ceramic resistor [15]. A high-speed camera (Phantom VEO 710) was used to record time-resolved images of the explosive dynamics. The sampling rate was no less than 220,000 fps, with an exposure time ~1 µs (per frame) and spatial resolution ~10^2^ µm. A lens was used to adjust the light emission of the explosion to a moderate level. The backlight source was a commercial 220 W-LED light (Jinbei EF-220) with a maximum luminance of Ln88000 Lux. Moreover, a commercial Fresnel lens (Jinbei ZF-6) was also used to focus the light spot to broaden it and make it more luminous. All waveforms were recorded by a Tektronix DPO 4104B (bandwidth of 1 GHz) digital oscilloscope. Synchronization of the high-speed camera trigger and wire explosion was made through the oscilloscope.

### 2.2. Products Collection and Characterization Methods

The wire exploded under liquid medium for approximately fifteen times, the produced nanoparticles after explosion formed a suspension with the liquid medium. Due to the great repeatability of electrical explosions and particle size distributions with a variable coefficient less than 4% [21], multi-repeated experiments to increase the amounts of explosive products had no influence on the final characterized results. The suspension was then centrifuged, and dried using a vacuum drying chamber, finally acquiring the nanopowders. Several methods were used to characterize the morphological and crystallographic characteristics, including SEM (Regulus 8230), TEM/HETEM (FEI Talos F200X G2), and XRD (Rigaku, Ultima IV). Therein, the XRD pattern of the sample was taken using Cu K_α_ radiation (λ = 0.15406 nm) at the working voltage and current of 40 kV and 40 mA, and the recorded range at 2*θ* was from 20° to 90° with a step size of 0.02°. Micromorphology of the sample was obtained by SEM and TEM at an accelerating voltage of 10 kV and 200 kV, respectively.

### 2.3. Experimental Methods

Experiments were performed with a single copper wire with a 50 μm diameter and 2 cm length that was exploded in different liquid media. The detailed experimental conditions are shown in Table 1. Therein, the stored energy can be calculated by *E* = 1/2*CU*^2^, where *U* is the applied voltage to the capacitor. The overheat coefficient (*ξ*), which can be expressed as the ratio of the final deposited energy to the energy for total vaporization (*ξ* = *E*_d_/*E*_v_), is commonly used to indicate the vaporized degree of the exploding wire. *E*_d_ is the deposited energy during explosion, as shown in Equation (2), and *E*_v_ is the energy needed for the total vaporization of the exploding wire (*E*_v_ is an inherent value for a certain load and is 2.2 J in our experiments). When *ξ* < 1, it means the wire is partly vaporized and the liquid drops are present, and the vaporized degree will be enhanced with increased *ξ*; however, total vaporization without liquid drops is almost impossible even at *ξ* > 1 due to the thermal and electromagnetic instabilities in EEW [22]. Moreover, because experimental parameters, such as the stored energy and media will influence the electrical explosion characteristics, as a result, the *E*_d_ and *ξ* will be different under diverse experimental conditions [1,20].

## 3. Experimental Results

### 3.1. Electro-Physical Characteristics, Bubble Dynamics, and Explosion Products of EEW in Liq N2

**Figure 2:**
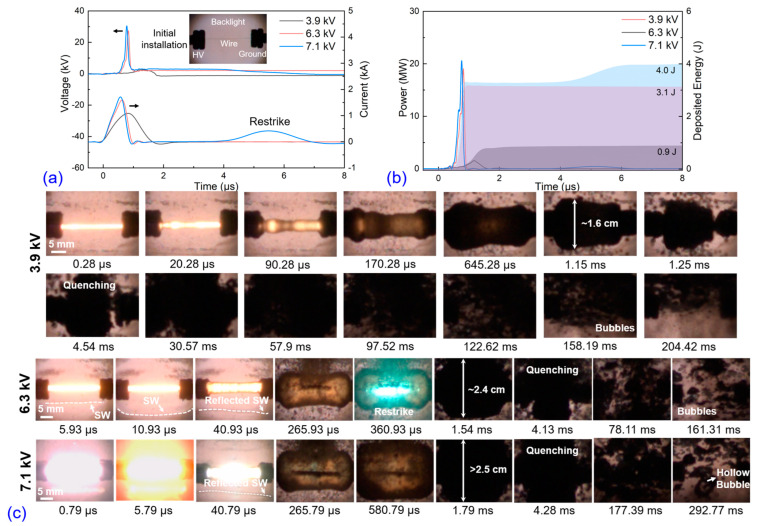
Copper wire electrical explosion in liquid nitrogen under three different applied voltages: (**a**) voltage and current waveforms, (**b**) power and deposited energy waveforms, and (**c**) corresponding high-speed camera images of wire explosion and later bubble dynamics.

The production of nanoparticles following electrical explosion is strongly influenced by the early discharge and later quenching processes, making electro-physical and dynamic analyses essential. Figure 2 illustrates the waveforms of voltage, current, power, and deposited energy, as well as the dynamics of the exploding wire captured by high-speed shadow images. In Figure 2a, the current rise rates increase from 1.3 to 2.9 kA/μs and with the increased applied voltages, the current peaks increase from 1.1 to 1.7 kA as well. Due to the insufficient stored energy in the 3.9 and 6.3 kV cases, the voltage between the electrode ends is not high enough to cause breakdown of the discharge channel (DC), and the current rapidly decreases to 0 within 0.83 and 0.37 μs. But in the 7.1 kV case, there exists a secondary current peak which is caused by the secondary breakdown when the expanded DC reaches a suitable *pd* value [15]. With the applied voltages increasing, the voltage peaks also increase from 2.5 to 30 kV. Typically, the moment of voltage peak is considered as the beginning of phase explosion and forms a highly resistive DC. Before this moment, the deposited energy is used to heat the wire and make the phase transition (from solid to vapor), but after this moment, the deposited energy is used to support the plasma process and it is consumed by optical and thermal radiations. In Figure 2b, it can be seen that the electric power boosts drastically from 1.5 to 20.5 MW. The deposited energy before voltage peak moment under the 3.9, 6.3, and 7.1 kV applied voltage cases is 0.7, 2.6, and 2.8 J, respectively, and the total deposited energy during discharge is 0.9, 3.1, 4.0 J. As the applied voltages increase, the overheat coefficients (*ξ*) are 0.4, 1.4, and 1.8 for the different cases, respectively. The 3.9 kV case (*ξ* < 1) exhibits incomplete vaporization, leading to the presence of un-vaporized liquid drops in the resulting products. However, the amount of un-vaporized liquid drops decreases significantly in the 6.3 kV and 7.1 kV cases. Nevertheless, it is known that achieving complete vaporization is extremely challenging due to thermal and magnetohydrodynamic instabilities, even with sufficient initial stored energy [22]. The corresponding high-speed shadow images of the exploding wire under the 3.9 kV case are shown in Figure 2c, providing a complete evolution of the process. Upon the injection of the pulse current, the DC rapidly expands and gives rise to an optical radiation burst due to the eruption of overheated and ionized matter. The expanding DC pushes the water away and creates a cylindrical bubble containing the hot metal vapor. At approximately 1.15 ms, the bubble reaches its maximum size with a diameter of 1.6 cm, after which it rapidly quenches within several microseconds. It is worth noting that multiple bubble pulses, which are common in electrohydraulic effects or electrical explosions underwater, are not observed [23]; inversely, after the first bubble is quenched, a large number of small bubbles are formed. As the applied voltage is increased, the discharge intensity is enhanced, resulting in more intense light radiation and DC expansion. The maximum diameter of the bubble increases from approximately 1.6 cm to more than 2.5 cm, and the average size of the quenched small-bubble groups increases from a sub-micrometer to a micrometer scale.

**Figure 3:**
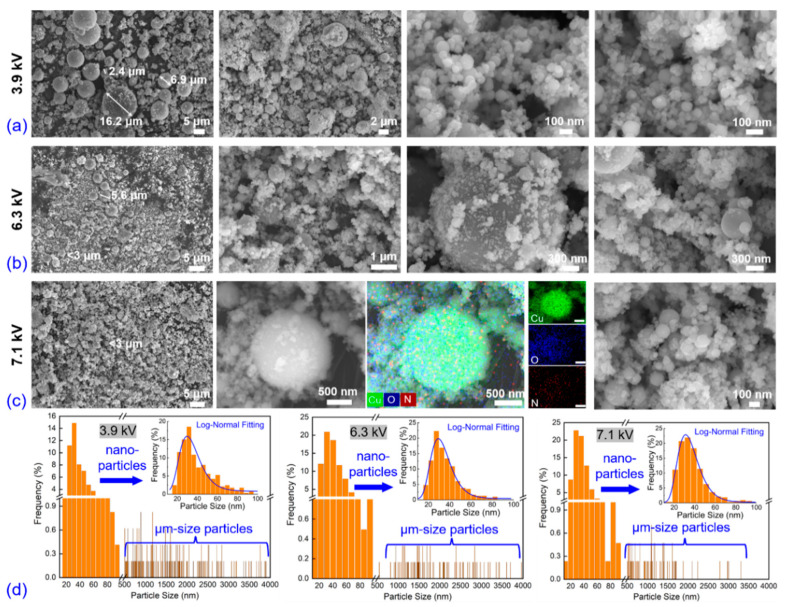
Morphology characterization via SEM and EDS equipment under three different applied voltages: (**a**) 3.9 kV, (**b**) 6.3 kV, (**c**) 7.1 kV, and (**d**) corresponding statistics of particle size distribution.

Figure 3a–c show the morphology and elemental distribution obtained via SEM and EDS equipment under three applied voltages. In all three cases, the explosive products consist of two characteristic spherical particle groups with typical dimensions of nanometers (<100 nm) and micrometers (several to a dozen μm), respectively. The first images in all three cases show a prominently decreased average size and ratio of micrometer-sized particles. The large-sized particles in the 3.9 kV case are believed to be due to un-vaporized liquid drops resulting from insufficient initial stored energy, whereas the dimensions and proportions of these particles decrease with increasing stored energy. In the 7.1 kV case, almost all particles are smaller than 3 μm. The nano-sized group is composed of spherical nanoparticles, which agglomerate with each other or adhere to the surface of larger particles, and the morphology is similar under all three voltage cases. The particle size statistics are presented in Figure 3d, and the inset shows the particle size distribution and fitting curve of the nanoparticles (<100 nm). The nanoparticle size distributions under all three voltages follow a common log-normal distribution, which is expressed by Equation (3) as follows:

(3) $$\begin{array}{l} f(d)=\frac{1}{\sqrt{2\pi }d\log\sigma_{g}}\exp\left\{-\frac{{\left(\log d-\log D_{50}\right)}^{2}}{2{\left(\log\sigma_{g}\right)}^{2}}\right\}\\ \log\sigma_{g}=\sqrt{\frac{\sum n_{i}(\log d_{i}-\log D_{50}{)}^{2}}{\sum n_{i}}}\\ \log D_{50}=\frac{\sum n_{i}\log d_{i}}{\sum n_{i}} \end{array},$$

where *f*(*d*) represents the log-normal distribution, *D*_50_ is the median diameter, *d* is the particle diameter, *n*_i_ is the number of particles with diameter *d*_i_, and *σ*_g_ is the geometrical standard deviation, respectively. The *D*_50_ under 3.9 kV, 6.3 kV, and 7.1 kV is 33.1 nm, 32.2 nm, and 34.3 nm, respectively, and the *σ*_g_ is 1.38 nm, 1.36 nm, and 1.35 nm, respectively. Surprisingly, the *D*_50_ value increased slightly with the increased initial stored energy, which is contrary to the usual results observed in air and water explosions [1,12]. This unusual behavior may be attributed to the quenching behaviors of the bubbles.

**Figure 4:**
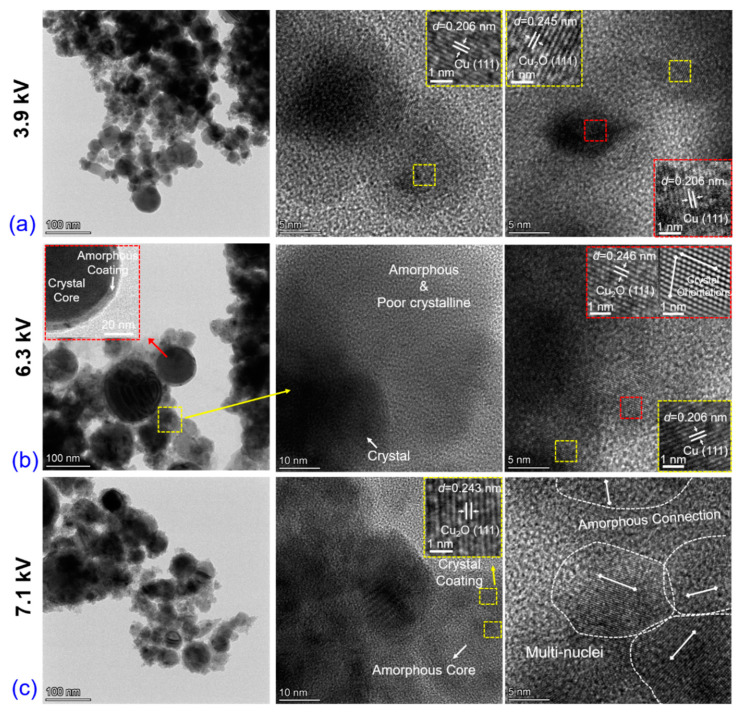
Morphology and crystalline characterizations via TEM/HRTEM equipment under three different applied voltages: (**a**) 3.9 kV, (**b**) 6.3 kV, (**c**) 7.1 kV.

TEM/HRTEM images provide further insight into the morphological and crystalline structures of the explosive products. Several characteristics can be observed under three voltage cases. Figure 4a illustrates that the crystal lattice distances, *d*, of the nanoparticles are 0.206 nm and 0.245 nm, corresponding to the (111) plane of the face-centered cubic Cu and Cu_2_O, respectively. In Figure 4b, the structure of the crystal core encapsulated by the amorphous shell can be observed, with the shell thickness typically ranging from 5 to 7 nm. The first image shows the presence of some light-color shapeless materials, in addition to the dark-color nanoparticles. Magnifying this area, as shown in the second image, reveals that these shapeless materials are amorphous or possess poor crystallinity. The structure of amorphous core-crystal shell is also observed, as shown in Figure 4c. The *d* of the shell is 0.243 nm, assigned to the (111) plane of the Cu_2_O phase. Furthermore, the light-color area corresponds to the aggregation of multi-nuclei connecting with the amorphous materials. The larger particles are the aggregation of numerous secondary fine nanoparticles, which were always observed in liquid media explosions [24].

**Figure 5:**
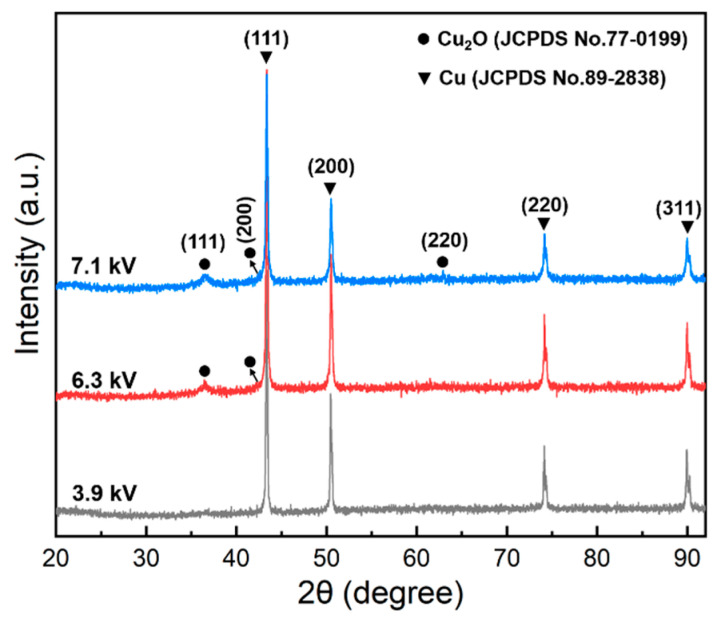
XRD patterns of collected copper powders after explosion under three applied voltages.

Figure 5 shows the XRD patterns under three voltage cases, revealing that the nanopowders consist mainly of the Cu and Cu_2_O phases, which is consistent with the HRTEM results. The narrow and intense diffraction peaks of Cu indicate its good crystallinity. The peak located at 36.5° corresponds to the Cu_2_O phase and becomes more pronounced at applied voltages of 6.3 kV and 7.1 kV. Additionally, the peak broadens as the applied voltages increase, indicating the presence of an amorphous Cu_2_O phase. Based on the HRTEM results, the amorphous shell and the light-color shapeless materials are likely to be amorphous Cu_2_O.

### 3.2. Electro-Physical Characteristics, Bubble Dynamics, and Explosion Products of EEW in Water

**Figure 6:**
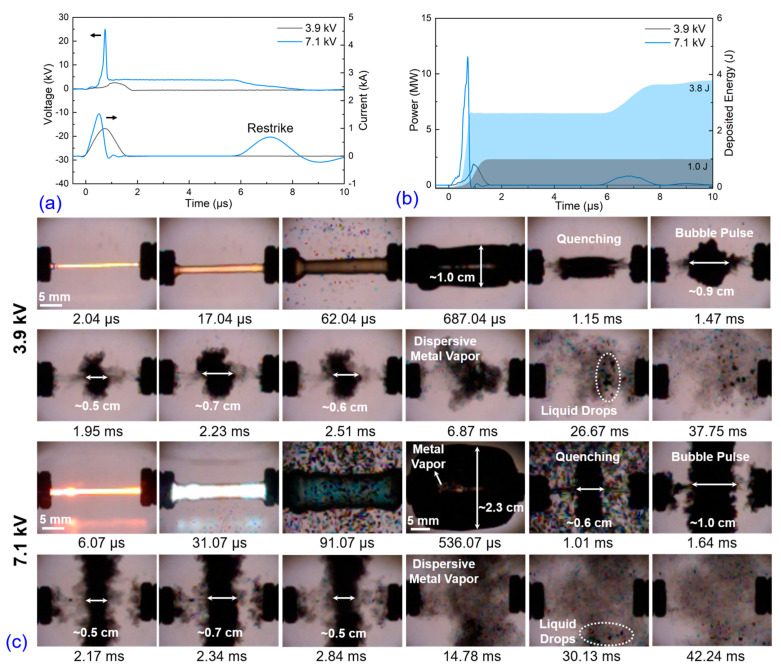
Copper wire electrical explosion in water medium under three different applied voltages: (**a**) voltage and current waveforms, (**b**) power and deposited energy waveforms, and (**c**) corresponding high-speed camera images of wire explosion and later bubble dynamics.

Water is commonly used as a liquid medium in EEW for preparing fine nanoparticles. In this study, an electrical explosion underwater was performed and compared with explosion in liq N2. The waveforms of voltage, current, power, and deposited energy are presented in Figure 6a,b. The waveforms in both cases are similar, indicating that the liquid media have a limited influence on the early discharge stage but mainly affect the vapor and bubble dynamic behaviors later on. The current peaks and current rise rates under applied voltages of 3.9 and 7.1 kV are 1.0 kA, 1.3 kA/μs, and 1.5 kA, 3.0 kA/μs, respectively. Identically, in the 7.1 kV case, there is a secondary breakdown. The voltage peaks are 2.8 and 24.9 kV, which appear at 1.1 and 0.7 μs, respectively. Correspondingly, the deposited energies before the voltage peak moment are 0.8 and 2.3 J, and the total deposited energies are 1.0 and 3.8 J. The overheat coefficients (*ξ*) are 0.4 and 1.7. According to the above waveforms and the parameters values, the electrical explosion characteristics are similar in water and liq N2; however, the bubble dynamics seem obviously different, which can be seen in Figure 6c.

In water explosions, former research indicates that the early cylindrical bubble is formed due to shock wave propagation and explosive product expansion [25,26]. The shock wave front propagates much faster than the bubble boundary, and the bubble is composed of the explosive products. After the collapse, the bubble not only further expands, but the explosive products transform into a cloud of particles that slowly diffuse into the surrounding water [27]. In Figure 6c, as shown in the shadow images after 100 μs, the first bubble reaches its maximum size at approximately 1.0 cm and 2.3 cm under 3.9 kV and 7.1 kV applied voltages, respectively. The hollow bubble contains hot metal vapors and quenches within a microsecond. Unlike the evolution in liq N2, the bubble has multiple pulses, which are called bubble pulses (BP). The BP occurs due to the rapid expansion of DC, compressing the water medium to form a bubble. As the bubble volume expands, the inner pressure decreases, causing the bubble to collapse when the external pressure exceeds the inner pressure or is destroyed by reflected shock waves. Due to the inertia force, the bubble experiences several pulses of expansion and shrinkage. After the final collapse of the bubble, the metal vapors come into direct contact with the water medium, which is different from the liq N2 case where the metal vapors are encapsulated by secondary small-size bubbles. In the 3.9 kV case, some un-vaporized micrometer-size particles are mixed with metal vapors, which correspond to the detection of large particles in the SEM results. Increasing the applied voltage reduces the amount and size of un-vaporized particles significantly.

**Figure 7:**
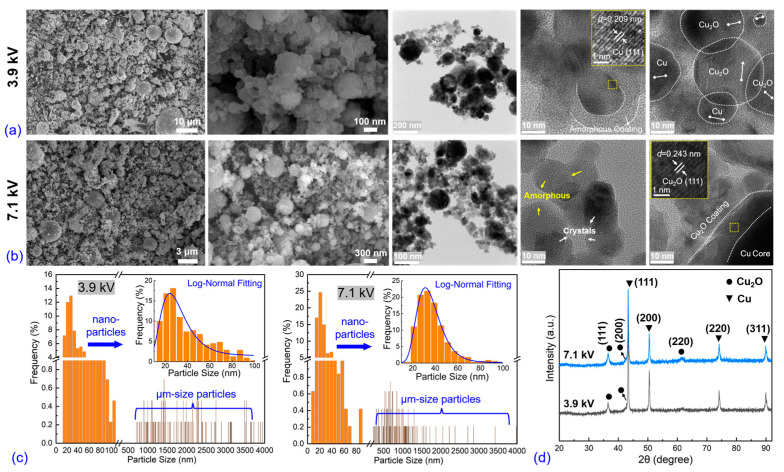
Morphology and crystalline characterizations via SEM, HRTEM, and XRD equipment under three different two voltages: (**a**) 3.9 kV, (**b**) 7.1 kV, (**c**) statistics of particle size distribution, and (**d**) corresponding XRD patterns.

Figure 7a,b depict the morphology and crystalline structures of the nanoparticles produced under 3.9 kV and 7.1 kV, respectively. The two typical dimensional particle groups, namely, the nanometer-scale and micrometer-scale groups, are formed, with the dominant group being the nanometer-scale particles as the applied voltage increases. Some nanoparticles present as polygonal rather than standard spherical, which is similar to the results reported by Krishnan et al. [19,28,29] and can be attributed to the final particle being composed of multiple nuclei, as shown in the last image of Figure 7a. Other characteristic crystalline structures are similar to the products formed in liq N2, such as the core-shell structure and a mixture of amorphous and crystalline materials. Figure 7c provides the particle size distribution underwater; similar to the products in liq N2 explosion, the products are also composed of nanometer-size (<100 nm) and micrometer-size particles. In the 3.9 kV case, the micrometer-size particles range from 0.8 to more than 4 μm; however, when increasing the applied voltage to 7.1 kV, the micrometer-size particle size mainly concentrates on the range of 0.3 to 1.5 μm. Identical with the liq N2 explosion case, increasing applied voltage will also decrease the micrometer-size particle size in the water explosion. The insets show the size distribution of particles with a size less than 100 nm, and the blue line is the fitting curve using log-normal fitting. The *D*_50_ under 3.9 kV and 7.1 kV is 29.2 nm and 22.2 nm, respectively, and the *σ*_g_ is 1.5 nm and 1.4 nm, respectively. The *D*_50_ significantly decreases with the increased applied voltage, showing the opposite trend to the liq N2 explosion but meeting the common results in EEW. This phenomenon may be attributed to the discrepant bubble dynamics. XRD patterns are also presented in Figure 7d, showing that the locations of the diffraction peaks are identical to the explosion in liq N2, and the products are composed of the Cu and Cu_2_O phases. The broadening of the peak at 60.5° to 62.6° corresponds to the (220) plane of the amorphous Cu_2_O.

## 4. Discussion

**Figure 8:**
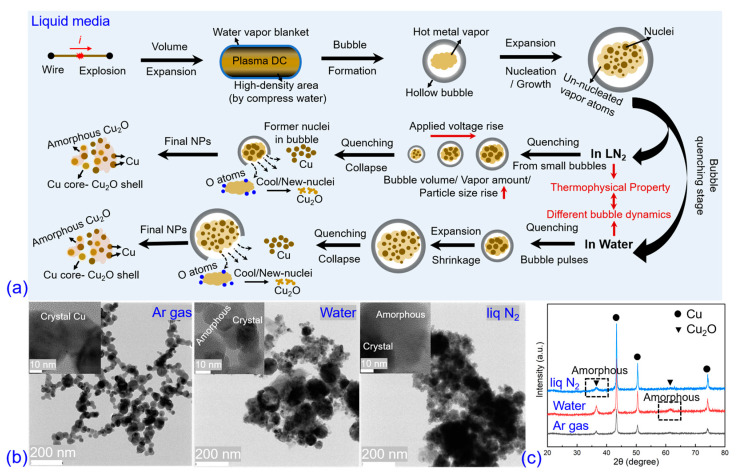
Schematic diagram of the nanoparticle formation mechanism in liquid explosion (**a**), TEM/HRTEM images of copper wire explosive products synthesized in Ar gas, water and liq N2 media, respectively (**b**), and the corresponding XRD patterns (**c**).

In the EEW process, nanoparticles are formed through a unique vaporization-condensation process, where the wire is overheated and rapidly quenched at an extremely high rate of ~10^9^ K/s, which has been verified by simulation [12] and experiments [30,31]. In gas ambient explosions, particle formation occurs through nucleation and growth mechanisms, while in liquid medium explosions, bubble dynamics play a crucial role in the process. Figure 8a illustrates the possible schematic diagram of the formation mechanism in a liquid explosion. When the pulse current goes through the metal wire, the intense Joule effect provides several hundred Joules of energy and heats the wire into an overheated metal vapor. Due to the drastic change in DC volume, the cylindrical DC pushes the water away. The internal energy of metal vapor transforms into the kinetic energy of the DC, rapidly expanding and evolving into a hollow micrometer bubble. Because of the energy conversion, the temperature of the metal vapor decreases. The vapor can also cool by collision with the gas molecules inside the bubble; however, unlike the free gas ambience, this collision-quenching mechanism is limited due to the finite space of the bubble. The decrease in vapor temperature reduces the saturated vapor pressure, and when the actual vapor pressure exceeds the saturated vapor pressure, nucleation begins. During this process, once the extremely high-temperature DC is in contact with the water, a thin water vapor blanket will form to isolate the DC from the water, which is the Leidenfrost phenomenon [20,32]. Relying on the influence of this effect, heat exchange (heat conduction and radiation) can be restrained within a short duration. In conclusion, besides a small number of vapor/drop cluster touches or traverses in the water interface, a considerably large amount of vapor atoms nucleate and grow inside the bubble, which is a mixture of liquid nuclei and un-nucleated vapor. Then, the bubble collapses (quenching stage) where the discrepancies between water and liq N2 explosion develop. The obvious distinctions of the bubble collapse behaviors in liq N2 and water should result from the difference in their thermophysical properties. Compared with room-temperature water, the matter properties (such as density, viscosity, saturated vapor pressure) of liq N2 possess extreme thermal sensibility, especially at the temperature near the boiling point. At the shrinking and collapse stages, the enhancement in heat transfer on the bubble surface leads the surface tension to decrease more than one order of magnitude of bubbles produced in liq N2 than in water, which may be responsible for the formation of the secondary fine bubbles in liq N2 [33,34]. In the liq N2 explosion, the main bubble collapses to numerous small-scale bubbles, and the dimensions of these bubbles rise with the increased applied voltage. The encapsulated amount of vapor is proportional to the volume of the bubbles, as well as the final particle dimension because of the particle growth through the collision of nuclei and the surface deposition of vapor atoms. This can explain why the *D*_50_ increases when applying a higher voltage. However, in the water explosion, the main bubble after multiple pulses collapses without the second small-scale bubbles, and the un-nucleated metal vapor directly enters into water. In this case, the vapor atom cluster quenches by collision with the ambient water molecules, nucleating and growing, which meets the common mechanism in EEW; therefore, the *D*_50_ decreases with the increased applied voltages.

The formation of amorphous Cu_2_O is also discussed simply. As the metal vapor inside the bubble is not completely exhausted, when the bubble finally collapses, the un-nucleated vapor atoms rapidly cool down in contact with the water, which leads to a considerable quenching rate and overcooling, providing the possibility for the formation of amorphous materials. This has been confirmed by previous research [35,36,37]. Figure 8b,c display the HRTEM and XRD characterizations of explosive products formed in gas and liquid media using the same wire load and approximately identical stored energy level (250–350 J). It indicates that the products formed in Ar gas explosions are well-dispersed spherical nanoparticles, whereas in liquid explosions, the products form aggregates composed of small-scale nanoparticles and connected amorphous materials. The greater thermal conductivity of the liquid medium leads to a higher quenching rate, which is considered a critical factor for the formation of amorphous materials.

## 5. Conclusions

Experiments were conducted to study the explosion of copper wire in liquid nitrogen (liq N2) under three different applied voltages. As a point of comparison, an explosion in water was also performed. The experimental results showed that the different liquid media had little influence on the early discharge stages but had a significant effect on the later bubble dynamics, which were characterized by the formation of secondary small-scale bubbles in liq N2, but multiple bubble pulses in water. Spherical pure copper nanoparticles and a small number of crystalline and amorphous Cu_2_O were obtained in both liq N2 and water explosions. With an increase in the applied voltage, the average size of nanoparticles in liq N2 increased from 33.1 nm to 34.3 nm. However, the trend was reversed in the water explosion, where the average size decreased from 29.2 nm to 22.2 nm. The discrepant bubble dynamics influenced the particle formation mechanism, including nucleation and growth processes.

## Figures and Tables

**Table 1:** Detailed experimental conditions and the crucial parameter.

Stored Energy (Applied Voltage)	Ambient Medium	Overheat Coefficient (*ξ*) ^1^	
		In Liq N2	In Water
1.5 J (3.9 kV)	Liq N2 and Water	0.4	0.4
4.0 J (6.3 kV)	Liq N2	1.4	/
5.0 J (7.1 kV)	Liq N2 and Water	1.8	1.7

^1^ Annotation: Overheat coefficient (*ξ*) is the ratio of the final deposited energy to the energy for total vaporization of the wire load.

## Data Availability

The data that support the findings of this study are available from the corresponding author upon reasonable request.
